# Self-perceptual blindness to mental fatigue in mining workers

**DOI:** 10.3389/fnrgo.2024.1441243

**Published:** 2024-10-23

**Authors:** Helena Purto, Héctor Anabalon, Katherine Vargas, Cristian Jara D, Ricardo de la Vega

**Affiliations:** ^1^Department of Psychiatry, Pontificia Universidad Católica de Chile, Santiago, Chile; ^2^AlertPlus, Santiago, Chile; ^3^Physical Education, Sport and Human Movement, Autonomous University of Madrid, Madrid, Spain

**Keywords:** fatigue, mental fatigue, reaction time, perception, fitness for duty, mining occupational health, occupational fatigue

## Abstract

Mental fatigue is a psychophysiological state that adversely impacts performance in cognitive tasks, increasing risk of occupational hazards. Given its manifestation as a conscious sensation, it is often measured through subjective self-report. However, subjective measures are not always true measurements of objective fatigue. In this study, we investigated the relationship between objective and subjective fatigue measurements with the preventive AccessPoint fatigue assay in Chilean mine workers. Subjective fatigue was measured through the Samn-Perelli scale, objective fatigue through a neurocognitive reaction time task. We found that objective and subjective fatigue do not correlate (−0.03 correlation coefficient, *p* < 0.001). Moreover, severe fatigue cases often displayed absence of subjective fatigue coupled with worse cognitive performance, a phenomenon we denominated Perceptual Blindness to fatigue. These findings highlight the need for objective fatigue measurements, particularly in high-risk occupational settings such as mining. Our results open new avenues for researching mechanisms underlying fatigue perception and its implications for occupational health and safety.

## 1 Introduction

Fatigue is a multidimensional phenomenon (Smith and Hale, 2007) whose precise origin is not yet well-elucidated (Behrens et al., 2023). A unified theory or definition of fatigue has not yet been attained. Nevertheless, it is a term that encompasses the decay in the ability to perform a task, where this may be accompanied by sensations related to the difficulty of the task at hand (McMorris, 2020). Central and peripheral fatigue may influence each other's manifestation (Amann, 2011), this fact gives us an insight on the level of complexity of fatigue, and therefore the challenges one encounters upon its study. Fatigue has been addressed at different explanatory levels: molecular, cellular, psychophysiological, and socio-ecological. Different models have been proposed to conceptualize fatigue. From a clinical and exercise physiology perspective, it's been delimited by two domains: fatigue perception and fatigability in performance (Greenhouse-Tucknott et al., 2022; Kluger et al., 2013). More complex psychobiological models have been proposed, where the conscious perception of fatigue is felt and processed as a feeling, related to a deviation from homeostasis (Noakes, 2012).

Beyond subjective fatigue feelings, neurobiology gives us hints on how different neurotransmitters and metabolites play a role in acute fatigue, such as the amount of catecholamines (Cools and Arnsten, 2022; McMorris, 2020) and glutamate (Möller, 2023) or adenosine (Davis et al., 2003) accumulation. Psychophysiology has given emphasis on the mind-body interaction that is mediated by the autonomic nervous system and its afferent and efferent branches (Amann et al., 2015; Behrens et al., 2023; Okano et al., 2015), where cognitive fatigue shows a distinct electrophysiological signature (DeLuca et al., 2009; Wylie et al., 2020), that is corresponded by autonomic tone changes (Junior et al., 2020). Socioecologically, fatigue emerges due to the interaction of the individual and its environment, where pollutants (Chandra et al., 2022), societal demands, circadian disruption (Meyer et al., 2022), lifestyle, among other factors, contribute to the development of fatigue (Karshikoff et al., 2017); the aforementioned factors also may influence the development of chronic fatigue, but physiologically this is a distinct phenomenon from acute fatigue. In this work we focus on acute occupational fatigue while recognizing these conditions share risk factors.

Occupational fatigue emerges as a multidimensional phenomenon, but also displays a fundamental principle throughout its different conceptualizations in different disciplines: the search for the greatest energetic efficiency. Physiology stipulates that an organism senses and responds to variations in its internal milieu and external environment (the anticipatory component) to maintain homeostasis; thus, one could think the conscious sensation of fatigue modulates behavior in order to preserve homeostasis, such as the sensation of thirst that emerges when blood osmolarity increases. A homeostatically regulated variable must be sensed in some way, thus, the sensation of fatigue could be part of a homeostatic system, where the regulated variable could be cognitive performance, degree of synaptic homeostasis or degree of function of certain neural circuits (where the set point indicates the optimal degree of performance). Nonetheless, up to this date, no clear sensor has been identified for these variables. In fact, the areas of the brain in charge of monitoring our performance and course correcting when errors are made, are precisely compromised during cognitive fatigue (Van Der Linden, 2011). The perception of performance decay during acute fatigue is a process that depends on brain regions that are precisely subject to fatigue. Furthermore, when fatigue is present, compensatory mechanisms that seek energetic efficiency may lead to mental heuristics, impulsivity in decision making, generalizations and ultimately mistakes and accidents (Mathew et al., 2018). These ideas suggest that feelings of fatigue may not align with objective psychophysiological markers, leading to what we term “fatigue blindness,” which is explored in this work. This underlines the importance of external fatigue detection in workers, especially in safety-sensitive tasks.

In the context of acute fatigue preventive measurements, we are ultimately compelled to understand its cognitive symptomology and its behavioral signature (i.e., reaction time), since this is easily measurable in a non-invasive manner (Jaydari Fard et al., 2019) and has a great impact on work safety. The appropriate completion of a task requires a degree of cognitive efficiency that is impaired during acute fatigue (Lorist et al., 2000). Mental fatigue is usually defined as a group of symptoms that emerge after a period of high cognitive demand (i.e., time on task effects) (Chaudhuri and Behan, 2000). A useful definition is “an executive failure to maintain and optimize performance over acute but sustained cognitive effort, resulting in performance that is lower and more variable than the individual's optimal ability” (Holtzer et al., 2010). Nonetheless, there is great variability on fatigue susceptibility despite the same cognitive load between individuals; this may be given by individual determinants such as genetic polymorphisms or personality. Therefore, it is hard to detect fatigue before the emergence of clear behavioral symptomatology such as delayed reaction time. Furthermore, fatigue is not a binary phenomenon, but presents itself in an intensity spectrum according to the degree of cognitive demand and the interaction with individual fatigability. Additionally, fatigability is influenced by diverse factors such as mental health, sleep quality, chronic disease, age, personality and lifestyle (Eldadah, 2010).

The neural mechanisms that underlie fatigue are yet to be understood, but it's been proposed to involve cognitive control mechanisms (Lorist et al., 2005), motivation (Dantzer et al., 2014; Müller and Apps, 2019) and monoaminergic pathways (Dantzer et al., 2014). Networks that are involved in motivated behavior control (the coordination between behavior and goals) change in their activity and connectivity during cognitive fatigue (Pezzulo et al., 2018). This includes highly communicated regions: the dorsolateral (dlPFC) and ventromedial prefrontal cortex (vmPFC), the dorsal anterior cingulate cortex (dACC) and the anterior insula (AI) (Dobryakova et al., 2013). During fatigue, a decrease in global integration of these networks is seen (Kok, 2022); this change in activity is sensed by structures that process interoceptive signaling (dlPFC, AI and dACC) that then influence motivated behavior (Kok, 2022). This impacts alertness and the ability to orient and sustain attention (Oken et al., 2006), which translates to a decay in performance in cognitive tasks that measure these domains (Krizan et al., 2023; Peters et al., 2022; Smolders and De Kort, 2014).

In the framework of decision making and cost-benefit analysis, fatigue emerges when the cost of mental effort is too high in relation to the predicted reward; the subjective cost of mental effort is discounted from the reward (Soutschek and Tobler, 2020). During fatigue, the cost of mental activity could be inflated. In this manner, subjective fatigue (emerging as an imbalance in cost-benefit that is subject to beliefs and mindsets regarding performance and abilities) could influence in objective fatigue parameters (such as reaction time) from a motivational standpoint. Thus, the subjective feeling of fatigue is subject to the energy availability estimates to be invested in a task, and predictions of the reward the task will bring; but it could influence task performance.

Despite the usefulness of viewing fatigue from a cost-benefit analysis, this approach inevitably incorporates subjective and individual factors (such as motivation) and fails to isolate or distinguish those variables from neurometabolic mechanisms that underlie fatigue. Furthermore, even when neurometabolic mechanisms are integrated with subjective ones, as in the Integrative Model of Effortful Control proposed by André et al. (2019), this approach doesn't consider why fatigue feelings may be dissociated from objective fatigue (presented in performance decrements), but rather proposes that feelings of fatigue emerge due to task costs. Given the aforementioned, we point to the importance of researching the frequent incongruence between fatigue feelings and objective fatigue. We propose that the awareness threshold of fatigue feelings is highly variable between individuals (in the same manner that interoceptive acuity and metacognitive abilities vary), giving rise to a phenomenon of self-perceptual blindness to fatigue. In this scenario, subjective fatigue feelings are dissociated from objective fatigue parameters (measured through psychophysiological or behavioral parameters). Recognizing the dissociability of subjective and objective fatigue is essential for its study because it acknowledges the diverse factors that influence both phenomena. Distortions in fatigue perception are analogous to differences in self-awareness regarding sleepiness, where there is discrepancy between objective and subjective measures of sleepiness.

Thus, fatigue is not simply an abstract feeling subject to motivational influences that may or not arise in consciousness. Rather, when these feelings correspond with the individual's fatigue level (as in fatigue acuity), one could argue the system has effectively conveyed synaptic milieu homeostasis information toward conscious awareness. Furthermore, lack of fatigue acuity (as in the self-perceptual blindness to fatigue we propose) can occur because this ability was not shaped evolutionarily (as in it has not been essential for survival).

When biological processes supporting synaptic homeostasis are surpassed by intense neural activity, that allostatic state could eventually increase the biological cost of activating certain neural circuits that are relevant to a particular task. This has been proposed to occur after prolonged cognitive tasks, where glutamate accumulation is seen in the dlPFC (Scholey and Apps, 2022). But persisting in a task despite decay in task performance is quite common; thus, factors influencing motivational control can override the ability to sense fatigue or self-monitoring of performance. Furthermore, the conscious feeling of fatigue is subjective in nature; it may be informed by physiological variables, but is also influenced by personality, for example Stephan et al. (2022). Thus, no failsafe has evolved to cease a task when performance decreases, rather, one could surmise that the system has evolved to continue engaging in the task; this could be achieved through compensatory mechanisms, such as an intercalation of on and off-task neural circuit activities. Activation of the default mode network during a task instead of on-task neural circuits could be a way of achieving synaptic homeostasis in on-task neural circuits, a sort of intercalated neural relay. If these off-task events occur in a safety sensitive task such as driving, a very new activity evolutionarily speaking, it could lead to accidents. Additionally, the estimations of ability to perform are given by fatigue-sensitive neural structures such as the dlPFC, inevitably leading to distorsions in predictions.

Thus, structures in charge of predicting the mental effort that a task requires, the reward it shall bring and sensing the present moment abilities are inevitably subject to environmental interference (such as metabolite accumulation). This implies that subjective monitoring of mental fatigue is not static, it may be dysregulated, such as exaggerations in present capabilities or in contrast a loss of self-efficacy given by different neurophysiological underpinnings that are yet to be elucidated. In this manner, subjective fatigue is not a good predictor of objective fatigue, because neural structures involved in interoception, metacognition and deployment of motivated behavior are altered in a previous cognitive effort magnitude dependent manner (McMorris, 2020).

Cognitive fatigue induces a decrease in attention and alertness (Holtzer et al., 2010). But it can also increase reaction times, a reflection of sensorimotor processing (Junior et al., 2020; Peters et al., 2022). One could argue that symptoms of cognitive fatigue emerge differentially regarding brain regionality, where decrements in more sophisticated cognitive domains (such as working memory) could appear before alterations in sensorimotor parameters. Nonetheless, reaction times are a validated behavioral parameter of fatigue, and delayed times are related to higher accident rates in tasks such as driving (Makishita and Matsunaga, 2008). This allows objective fatigue measurements.

Mining work implies various conditions that favor occupational fatigue development, particularly, circadian disruption (due to shift work), exposure to environmental pollutants, vibrations, extreme climate, and low atmospheric pressure. Given the subjective nature of fatigue-perception, external monitoring and detection of fatigue is essential for preventing work hazards and accidents. AlertPlus, together with Austrian technology (Sturm, 2012), has developed an optimal way of measuring objective fatigue that has been implemented for more than 10 years in Chilean mining workers (Anabalon et al., 2016). This present work aims to describe the importance of objective fatigue measurements in Chilean mining workers and evaluate if subjective fatigue interacts and/or influences objective fatigue.

In this study we measure objective fatigue through decrements in intrinsic alertness. Nonetheless, these are not only explained by mental fatigue, but also other variables such as circadian rhythms, quality of sleep, use of hypnotic medications and chronic conditions. In this study we do not control for those variables, but rather focus on a fitness for duty assessment, where we conceptualize this as measuring occupational fatigue in the real world. Fatigue is a multifactorial phenomenon, there is no gold standard for its measurement. Here we propose an ecological construct of fatigue that encompasses the influence of variables such as stress and sleep disruptions, while recognizing that in laboratory settings mental fatigue is induced and these variables are ideally controlled. This allows recognition of the fact that preventive fatigue measurements (before a work shift) are essential for work-safety, and that in the real-world fatigue is a phenomenon that cannot be isolated from the influence of time of day or sleepiness. Thus, when we refer to fatigue in this research, we refer to the construct of occupational fatigue encompassing other states that influence intrinsic alertness.

Mental fatigue implies less cognitive control, this translates to impulsive decision making and reduces capacity to perform certain mental tasks. Objectively it is measured as a decrease in task performance; subjectively it is reported as a feeling of mental tiredness. Nonetheless, self-perception of cognitive status is given by interoceptive, and metacognitive abilities vary between individuals. Regions in charge of interoception and metacognition change in their activity during fatigue states (Chen et al., 2020; Maniscalco et al., 2017). Given the preceding evidence, we hypothesize that subjective feelings of fatigue do not correlate with objective fatigue measurements. In this work, we also propose a useful concept for studying occupational fatigue: the phenomenon of perceptual blindness to fatigue, given by the discordance between acute mental fatigue subjective self-report and acute fatigue objective measurements. During this phenomenon, the worker reports being apt for work despite presenting acute fatigue.

## 2 Materials and methods

This descriptive and correlational study was done using AccessPoint system acute fatigue measurements in Chilean mining workers from the year 2019 to 2023. Workers from 61 mine sites were included.

### 2.1 Participants

This study involved the participation of 680 females (average age 40.4 ± 8.41) and 11,965 men (average age 46.8 ± 10.9). With an average of 39.2 ± 95.1 measurements per individual; 481,932 measurements in men and 14,328 measurements in females. The workers in this study are subject to annual pre-occupational screenings, which include a physical health check and a rigorous psychometric assessment with Vienna Test System.

### 2.2 Measurement tools

AccessPoint system was used for acute occupational fatigue estimation; this system includes a questionnaire that measures subjective fatigue (Samn and Perelli, 1982) and a neuropsychological test named WAFA (Sturm, 2012) that evaluates alertness level and attention through reaction time. The WAFA test involves the presentation of a visual stimulus on a screen (a black circle) at variable intervals of 2–10 s; before the task, the worker is instructed to press a button as soon as the stimulus is present. Each fatigue measurement includes a self-report of waking hours. The evaluation lasts 2 min.

### 2.3 Procedure

Before the start of the workday, each individual completed a 90 s evaluation in an AccessPoint apparatus on site (Anabalon et al., 2016).

### 2.4 Measurements

#### 2.4.1 Sustained attention and objective fatigue

Sustained attention was quantified according to the WAFA test (Sturm, 2012), that measures the cognitive domain of attention, particularly alertness level and psychomotor vigilance (Gaurav et al., 2021). WAFA test score is calculated according to reaction time to visual stimulus and the number of successful trials, where the worker can get a WAFA score of 0–100; severe fatigue is defined for scores below 20 points (Gaurav et al., 2021). The software by Schuhfried company provides a WAFA score from 0 to 100, allowing classification and presence of occupational fatigue. The scoring method is built upon reaction time across trials, the exact formula is confidential as it belongs to Schuhfried company that provides the cognitive task. A score below 20 indicates severe fatigue; between 20 and 40 moderate fatigue; 40–60 slight fatigue; 60–100 absence of fatigue. All cases of moderate and severe fatigue (score of 0–40) were classified as having objective fatigue. This task has been convergently validated with other well-established lengthier tasks that assess mental fatigue such as the Stroop task or psychomotor vigilance task through time on task effects. This task allows fatigue detection in a short time window given the higher cognitive load it possesses. Longer versions of the task have been used in previous studies, but their applicability on terrain with real life workers was not operationally successful.

#### 2.4.2 WAFA task

This task measures short-term intrinsic alertness (endogenously controlled) to a visual stimulus. This variable (Intrinsic alertness) is the logarithmic mean of the individual reaction times. There are subsidiary variables in this task: Median reaction time; Dispersion of reaction time (the exponent of the standard deviation of logarithmic reaction times); Number of missed reactions (number of stimuli to which no response was made within 1,500 ms); Number of false alarms (number of times a response key was pressed when no stimulus had been presented). The reliability coefficient (Cronbach's alpha) of the WAFA task in the short form (used in this study) is 0.947; Weighted omega = 0.905. Test results are interpreted in percentile ranks (PR), from there, it is possible to determine if the worker's score is above average, average, or below average. In the area of occupational health, a broader average range is used (16–84). A 0–15 PR in the WAFA task is defined here as below average, providing a way of defining a state of occupational fatigue.

#### 2.4.3 Subjective fatigue

Fatigue self-perception was measured with the Samn-Perelli scale that goes from 1 to 7 and asks: “How are you feeling now?”. 1 = “Full of energy”; 2 = “Very good”; 3 = “Good”; 4 = “A little tired”; 5 = “Moderately tired”; 6 = “Very tired”; 7 = “Completely exhausted”. Scores of 4–7 have were classified as positive for subjective fatigue. Scores from 1 to 3 were classified as negative for subjective fatigue.

#### 2.4.4 AccessPoint Risk Matrix

AlertPlus identified a discordance between objective (WAFA test) and subjective (Samn-Perelli) fatigue measurements. As a result, AlertPlus operationally defined four categories to capture the relationship between subjective and objective fatigue: “Stable” (neither subjective nor objective fatigue), “Tension” (subjective fatigue only), “Distress” (objective fatigue only), and “Eustress” (both subjective and objective fatigue). Although these terms have specific meanings in psychology, we retained them for consistency with AlertPlus' internal terminology.

The “Distress” terminology was chosen because this population could be at a higher risk of endangerment, given by the fact that the worker subjectively reports not being fatigued. The “Eustress” terminology, even though its meaning relates to positive stress, we chose this denomination because it is a more adaptive response to fatigue than the scenario of blindness (Distress). “Stable” terminology was chosen because at the time of measurement the worker does not present any type of fatigue. Finally, “Tension” terminology was chosen because this word is used to describe states of higher nervousness, which we hypothesized could be the case of workers overestimating their degree of acute fatigue. This classification allowed assessment of differences in cognitive task performance between different fatigue states. Importantly, subjective fatigue is measured through the Samn-Perelli scale and objective fatigue through the WAFA Test.

### 2.5 Statistical analysis

R software was used for statistical analysis (R Core Team, 2021). To evaluate an association between objective and subjective fatigue measurements, a Pearson correlation test was done between the Samn-Perelli scale results and the WAFA test score. For assessing objective fatigue differences between males and females, mean WAFA score was compared with a *t*-test. The frequency of Distress was assessed in the population with an WAFA test score below 20 (indicating severe acute fatigue); a proportion analysis for one sample with continuity correction was employed to compare Distress frequency with the other categories. Finally, mean WAFA Test was compared between the different AccessPoint Risk Matrix categories with an ANOVA test and then a Tukey test; effect size was measured with a Cohen's *d*-test.

## 3 Results

### 3.1 Participants

Most of the population (*n* = 12,645) were men (*n* = 11,965, 95%). Mean age was 46 years, in a range between 18 and 78 years (Table 1). A total of 496,314 measurements were done, with 481,932 in men and 14,382 in women.

**Table 1:** Study population.

**Variable**	***n***	***%***	**Mean**	**SD**	**Min**	**Max**	**Med**	**IQR**
**Age**								
Below 19 years	146	0.03	18.64	0.50	18	19	19	1
20–29 years	20,083	4.03	27.33	1.54	20	29	28	1
30–39 years	114,535	23.01	35.01	2.77	30	39	35	5
40–64 years	347,254	69.77	50.65	6.72	40	64	50	11
65 and up years	15,703	3.15	67.18	2.30	65	78	67	4
**Gender**								
Male	11,965	94.55	46.81	10.19	18	78	47	16
Female	680	5.45	40.42	8.37	18	77	39	14

The number of people is described by age range and gender (*n*). Then, the average, standard deviation (SD), minimum value (Min), maximum value (Max), median (Med) and interquartile range (IQR) of the age of each age range and gender.

### 3.2 Neurocognitive measurement

AccessPoint assessment included the neuropsychological WAFA Test (score from 0 to 100); average score in men was 44.5 (SD 19.0, minimum 0, maximum 99, median 43). For females, average score was 41.9 (SD 19.2, minimum 0, maximum 98, median 40).

### 3.3 Subjective and objective fatigue

**Figure 1:**
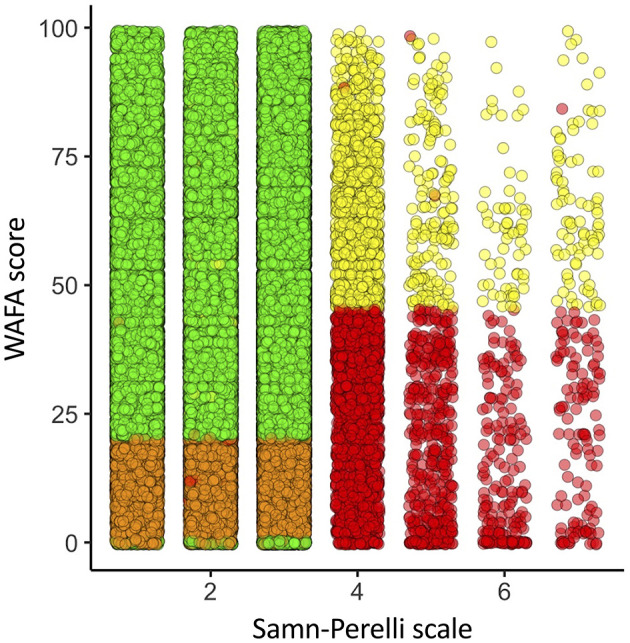
Correlation between WAFA score and subjective fatigue. Pearson correlation between Samn-Perelli scale (subjective fatigue; 1–7) and WAFA score (objective fatigue measured with WAFA test 0–100). Correlation coefficient value: −0.03. *P*-value = 1.3e-77, *p* < 0.001. Eta square value = 0. Each point represents a different measurement. Stable in green; Tension in yellow; Distress in orange; and Eustress in red.

A Pearson correlation test was performed between subjective fatigue (SF) and objective fatigue (OF) across all AccessPoint measurements (males and females), yielding a correlation coefficient of −0.03 and a *p*-value of 1.3e-77 (Figure 1). This indicates no significant correlation between the two measures of fatigue, with the extremely low *p*-value likely attributable to the large sample size. An Eta Squared test was also conducted, and as expected given the correlation results, a value of 0 was obtained, suggesting that the variance in WAFA Test scores cannot be explained by the variance in SF.

### 3.4 AccessPoint between males and females

A *t*-test for independent samples was done to compare mean WAFA Test scores between males and females; where males obtained a significantly higher mean score (44.73) than females (41.92), with a *p*-value of 1.06e-66 and a t value of 17.34.

### 3.5 Objective fatigue and AccessPoint Risk Matrix

**Figure 2:**
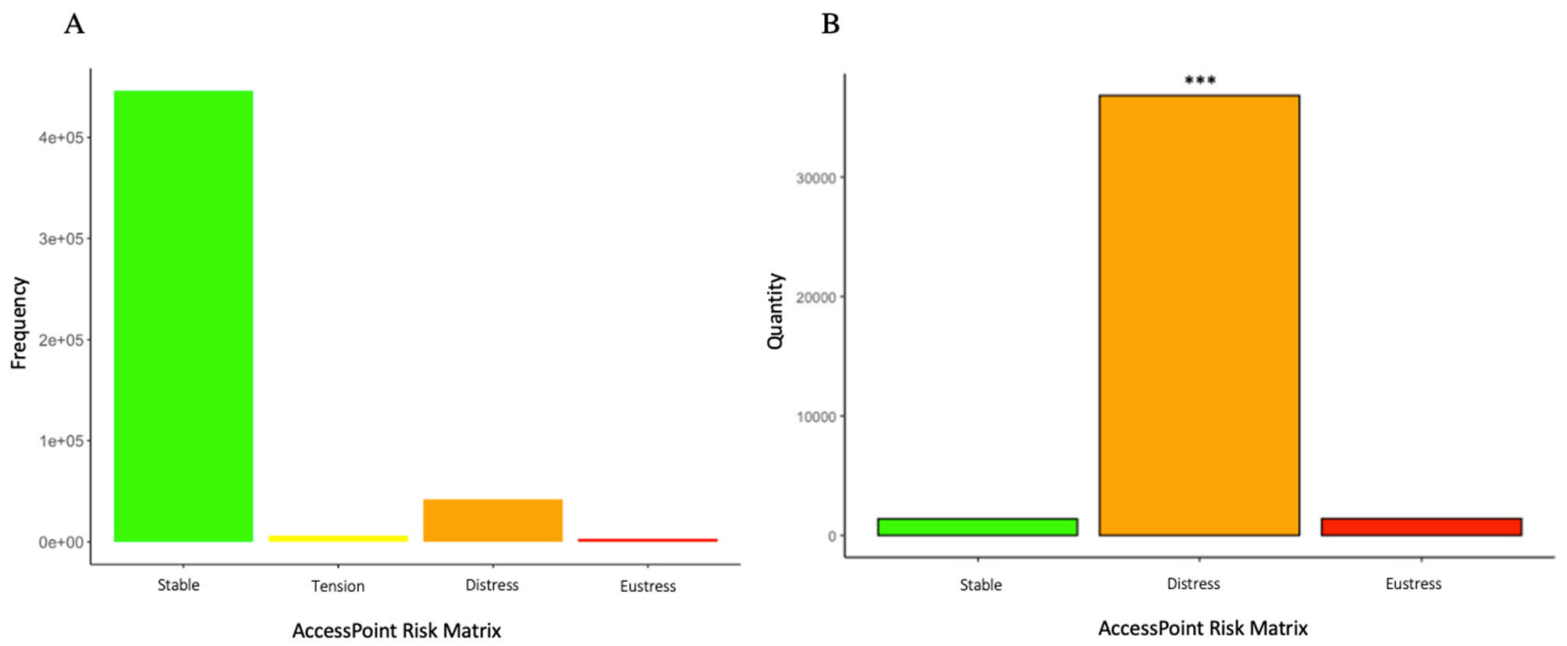
AccessPoint Risk Matrix frequency. In **(A)** AccessPoint Risk Matrix result frequency for all measurements. **(B)** AccessPoint Risk Matrix frequency for severe objective fatigue measurements (WAFA Test below 20). Proportions test for one sample with continuity correction compared the frequency of Distress in relation to the rest of the variables, indicating significant differences (*p*-value < 2.2e-16; X-square 449046; IC 95% for the true proportion: between 0.93 and 1.0). ***p < 0.001. Stable in green (absence of fatigue); tension in yellow (presence of subjective fatigue, absence of objective fatigue); distress in orange (absence of subjective fatigue, presence of objective fatigue); and eustress in red (presence of both objective and subjective fatigue).

The AccessPoint (AP) Risk Matrix allows to operationally define if SF coincides or not with OF. To assess the frequency of the AP risk matrix, total AP measurements were graphed (Figure 2A), where most measurements were Stable (445,930), followed by Distress (42,029), then Tension (6,620) and finally Eustress (3,143).

To find out if SF was present in the population with OF, we selected measurements with an RT Test score below 20 (severe OF); the count of each result of the AP Risk Matrix was graphed (Figure 2B). The presence of Distress is of great importance in the context of work safety, since the person reports not having fatigue despite being objectively fatigued; because of the aforementioned, we sought to evaluate if the frequency of Distress was significantly different from the frequency of other states within the severe fatigue group (RT Test score below 20). The proportions test for one sample with continuity correction indicated that there is a significant difference in the proportion of the category Distress in relation to other categories, with a *p*-value below 2.2e-16 and an X square value of 449,046. Thus, self-reporting absence of fatigue was more frequent than self- reporting presence of fatigue within the severe fatigue group.

### 3.6 Comparing WAFA test score between AccessPoint Risk Matrix categories

**Figure 3:**
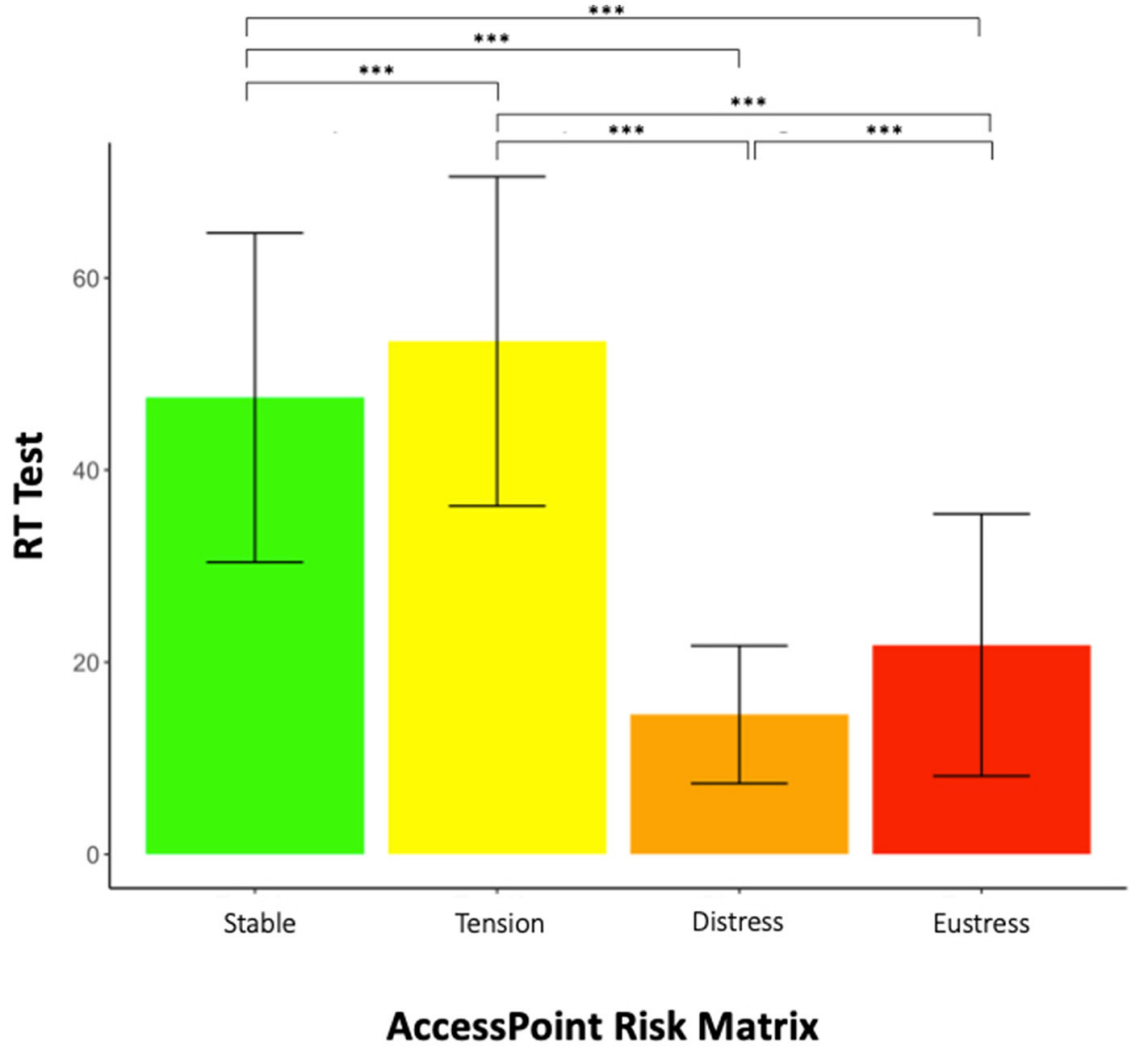
RT Test score and AccessPoint Risk Matrix. Mean and standard deviation of the WAFA Test is shown for each Risk Matrix classification. A Tukey test was done: *p* < 2e-16. Effect size for the comparison between categories was calculated through Cohen's *d*-test. Between Eustress and Distress there was a small effect size (*d* = −0.34); for Tension and Stable, we found a big effect size (*d* = 1.51); for Eustress and Tension, we found a big effect size (*d* = 1.99); for Distress and Stable *d* = −1.96; Eustress and Stable we found a big effect size *(d* = 0.93). ***p < 0.001.

In order to visualize possible differences in cognitive task performance between groups with and without subjective fatigue, the mean and standard deviation of the WAFA Test was graphed for each one of the AccessPoint Risk Matrix categories (Figure 3). In order to assess WAF test score differences between categories, we initially did a one-way ANOVA, where we detected significant differences in mean WAFA Test score between Risk Matrix groups (*p* < 2.2e-16; *F* = 53,804). We then did a multiple comparison analysis with a Tukey test, where we found significant differences in WAFA Test score between all groups (Distress, Eustres, Stable and Tension) with a *p*-value < 2.2e-16 for all comparisons. We calculated effect size with Cohen's *d*-test, indicating a big effect size (above 0.9) for all comparisons, except the comparison between Stable and Tension categories, where we found a small effect size (*d* = −0.342). The Eustress group (with OF, without SF) had a higher RT test score than the Distress group (with OF, without SF); while the Tension group had a higher score than the Stable group. In summary, these results indicate that perceptual blindness to fatigue (Distress) implies a higher degree of objective occupational fatigue (Distress groups have a worse RT Test score than Eustress group), and that the presence of SF not necessarily implies that there is OF (Tension).

## 4 Discussion

### 4.1 Main findings

The present study measured acute occupational fatigue with the AccessPoint (Anabalon et al., 2016) system in a Chilean mining worker population. We sought to study the relationship between SF [measured through Samn-Perelli scale (Samn and Perelli, 1982)] and OF [measured through the WAFA test (Sturm, 2012)]. We found that SF and OF do not correlate. This suggests that SF measured through self-report is not a good measure of OF, in other words, fatigue self-perception is not a trustworthy measure of fatigue measured through reaction time to a visual stimulus. In this manner, we confirm our initial hypothesis, and our findings add to the body of evidence suggesting OF and SF do not correlate (Cockshell and Mathias, 2014; Mehta et al., 2017; Peng et al., 2022), but are rather independent phenomena that are not good predictors of each other. Operationalizing the relationship between objective and subjective fatigue through the AccessPoint Risk Matrix allowed identification of differences in cognitive performance in relation to fatigue self-perception. Where the Distress group (with SF, without OF) presented the lowest score, followed by Eustress, then Stable and finally Tension (presenting the highest cognitive performance in the task). These different scenarios we detected again suggest that SF is not a good measure of OF.

### 4.2 Distress

Operationally, Distress was defined as the presence of OF without SF. This group is highly relevant in the context of work-safety, since not being able to sense the lack of mental and physical aptitude to perform tasks and instead believing one is fit (and therefore proceed to initiate or persist in a task), may lead to accidents (Namian et al., 2021). In our study, individuals with severe occupational fatigue (WAFA Test score below 20) rarely reported SF, suggesting a vulnerability to not perceiving or reporting SF. We propose that a higher degree of occupational fatigue leads to more self-perceptual blindness to the real degree of cognitive fitness to perform a task. This scenario is in line with the evidence that sustains this work's hypothesis, since functions such as conscious perception of fatigue and decision making rely on fatigue-sensitive brain regions (Arnsten and Shanafelt, 2021; Cools and Arnsten, 2022). Further research could help elucidate this putative threshold of fatigue where loss of ability to report (or metacognitively detect deterioration in performance) occurs in the fatigue spectrum; but possibly this threshold is highly variable between individuals and is subject to many factors.

This perceptual blindness we propose is in line with current stances on the neurobiology of fatigue, such as the Allostatic Self-efficacy Theory (Stephan et al., 2016) that illustrates how the feeling of fatigue emerges due to a mismatch between the sensing of bodily homeostatic states and ability to return to homeostasis. This theory states that perception of fatigue occurs when internal models generated on bodily homeostatic states do not coincide with the current states; the system calculates it doesn't have enough control or efficacy to modulate the bodily state. This is a useful framework for understanding the emergence of fatigue in disease states such as depression but may also be applied for the lack of subjective fatigue during objective acute fatigue. In that manner, fatigue feelings seem to depend on individual interoceptive acuity and metacognitive ability (to monitor quality of cognition or level of alertness for example); two processes that are susceptible to distortion (St Clair Gibson et al., 2003). This is illustrated in two misperception scenarios: during exaggeration of fatigue perception despite being fit for a task; or on the flip side, fatigue blindness, i.e., believing one is fit for a task in spite of present occupational fatigue. Future research could help elucidate what factors influence interoceptive and metacognitive abilities and therefore contribute to the degree of perceptual blindness to fatigue.

Further evidence supports this notion of fatigue perceptual blindness. During cognitive fatigue states (an aspect of occupational fatigue), the prefrontal cortex (in charge of metacognitive abilities) is highly sensitive to decay in function (Arnsten and Shanafelt, 2021). Furthermore, the degree to which cognitive fatigue interferes with brain function is dependent on regionality, where more recently evolved brain areas are early responders to fatigue. More research is needed to understand these differential effects of cognitive fatigue. Nevertheless, the inner monitoring of cognitive processes (for example, subjectively perceiving a slowing down of thought or greater distractibility) is highly impeded during fatigue (St Clair Gibson et al., 2003); explaining the loss of acuity in sensing if one is fit for a task during fatigue states (analogous to the extreme scenario of drunk drivers claiming to be fit for driving). The latter scenario reflects a loss of risk assessment abilities in decision making; but one cannot omit the context in which these measurements are made (usually before the workday), where possibly the pressure to work more hours (or avoid the consequences of absenteeism) may lead to a mismatch between reported and real subjective fatigue. Independent of the origin of this perceptual blindness we detected, these findings point to the inadequacy of subjective measures in fatigue detection. Additionally, measuring fatigue in the real world (where one cannot control for many variables) grants ecological validity and enriches our understanding of fatigue. Future research could include more in-depth questionnaires that evaluate different aspects of fatigue perception, such as self-efficacy.

### 4.3 Subjective and objective fatigue

Some researchers suggest that objective changes in task performance are the most relevant fatigue measurement (Holtzer et al., 2010); while others propose that SF is an informative or adequate measurement (Holtzer et al., 2016; Peters et al., 2022); nevertheless, most evidence suggests that there is no relationship between both phenomena (Cockshell and Mathias, 2014; Sandry et al., 2014; Völker et al., 2016). The present work observed the latter. Still, more studies are needed to confirm that SF is a bad predictor of OF. But one cannot omit the possibility of SF influencing OF. One could evaluate in a longitudinal manner if SF perception influences future OF development; we propose this since subjective fatigue sensations sometimes precede changes in cognitive task performance (Kanfer, 2011).

### 4.4 Fatigue spectrum

Despite the great clinical relevance of fatigue perception in absence of objective fatigue (Tension group), in this work we are compelled to support work safety. In that sense, it's more critical for worker safety when the individual doesn't report fatigue in spite of its presence. This previously mentioned phenomenon of fatigue “blindness” reflects the neurobiological mechanisms that underlie fatigue; where the capacity to perceive it and make adequate decisions (desisting from a task, taking a break, or reporting the presence of it) is hindered. Differences in subjective fatigue self-report suggest severe fatigue development goes in parallel with reduced ability to perceive or report fatigue. The presence of acute severe fatigue leads to a decay in mental activity, where heuristic processes (or mental shortcuts) are favored. This generalization may lead to a higher degree of mistakes in tasks that demand high alertness and attention (Mathew et al., 2018).

### 4.5 Subjective fatigue

SF perception is highly relevant in clinical scenarios and athletics, where this perception influences task performance (be it in daily life tasks or a stress test). This last point opens another relevant sphere around fatigue: motivation. Task persistence and performance in a task are related to motivation (Müller and Apps, 2019). This has been evident in exercise and fatigue studies, where the dACC, anterior insula and lateral PFC have been shown to be involved in motivating effort output and internal state processing (Müller and Apps, 2019).

Interestingly, we observed that the Tension group had the best performance in the WAFA Test. Perhaps, in Tension, fatigue perception (be it for a nascent fatigue that is not manifested yet in reaction time or simply underestimating abilities) possibly leads to early compensation (for example autonomic tone changes) that could favor higher reaction time and acuity in the task (Finke and Schächinger, 2020). Another possibility is the presence of higher neuroticism in this population [a personality trait that favors development of fatigue (Stephan et al., 2022)]; this may lead to greater sensibility to slight changes in cognition and physiology, which may explain reporting fatigue in absence of it. Furthermore, our present task may not be sensitive yet to the possibly real fatigue these individuals feel (as in, there is fatigue, but it is not affecting reaction time). Tests that evaluate other cognitive domains could be useful. It is essential to keep in mind that the cognitive tasks used to evaluate fatigue, although useful given their short duration, will not necessarily fully reflect performance in more complex day-to-day tasks, but given their narrow evaluation window and limited domains of cognition evaluated, aspects of the fatigue phenomenon will inevitably be sacrificed.

Although we do not yet understand how SF influences OF, there is strong evidence that mental processes (such as beliefs or mindsets) influence physiological ones, and vice versa; that is, the belief about one's own performance influences it. Cognition is embodied (Varela et al., 2017), and mind body connection is a tangible fact (Littrell, 2008). An evident example of this are behavioral changes during sickness (Dantzer and Kelley, 2007; Lasselin, 2021) or autonomic changes that occur during imagined or anticipated movement (Collet et al., 2013). This bidirectional mind-body interaction (Azzalini et al., 2019) suggests that SF is not something one must omit during fatigue prevention, because even if the SF is real or not, the lived experience of the individual eventually could influence performance. Maybe this fatigue feeling is not reflected in reaction times but may influence complex tasks. Thus, the value of these “fatigue feelings” cannot be underestimated, since in the absence of external tools, this is the way par excellence that will allow the system to be alerted to internal disturbances. For now, we need to better understand the mechanisms in charge of conscious fatigue perception. This knowledge could help improve fatigue perception acuity.

### 4.6 Biological sex differences

Females displayed a significantly lower WAFA Test score than males, this suggests a higher reaction time in females, or possibly a higher frequency of objective fatigue; nevertheless, ideally a balanced sample is needed, more OF tests (evaluating other cognitive domains); standardizing for variables that may influence fatigue development such as sleep hygiene habits or circadian health. Sex differences in fatigue development have been reported (De La Vega et al., 2022), but more studies are needed.

### 4.7 Fatigue perception, proposed mechanisms, and evolutionary perspective

The influence of cognitive fatigue on perception has been reported in sports performance, where cognitive fatigue distorts effort perception in the athlete (McMorris, 2020) and fatigue perception limits performance (Greenhouse-Tucknott et al., 2022). Although exercise is a predominantly motor task, regions in charge of evaluating fatigue (comparing internal bodily state with models generated upon previous interoception) during exercise, meaning, integrating information to decide effort output, are the same regions involved in cognitive fatigue perception. Particularly, the PFC, ACC and anterior insular cortex (AIC) are involved in fatigue perception during exercise; they integrate sensory information, modulate motor output and volitional movement (Robertson and Marino, 2015). The ACC and AIC are hubs of the salience network, assessing inner bodily states, seeking to predict perturbations and future threats to homeostasis; this allows anticipatory behavioral changes that minimize changes in homeostatically regulated variables (Hilty et al., 2011). The aforementioned regions are involved in cognitive fatigue perception. The dorsolateral PFC (dlPFC), vmPFC, dACC, insula and striatum are involved in cognitive fatigue (Peng et al., 2022; Wylie et al., 2017). The insular cortex and ACC are involved in various cognitive tasks that demand mental effort (Engström et al., 2015). The fact that the ACC and dlPFC are involved in conflict detection and cognitive control implementation (Parris et al., 2009) and at the same time are activated to implement avoidant response strategies (Hofmann et al., 2012), indicates that they play a role in energy preservation. This hints to common mechanisms between cognitive and physical fatigue.

During a physical task, decreasing performance when close to failure is key for physical integrity preservation; nevertheless, regarding cognitive performance, given our survival depends on our cognition (and proper interaction with the environment), possibly there are mechanisms that favor persistence rather than ceasing, regardless of decay in performance. This ability to persist in a task despite decay in cognition makes sense from an evolutionary perspective, where there are mechanisms in place that allow persistence in task (given the importance of a task and available resources to devote to the task) such as increased catecholamines (Cools and Arnsten, 2022). Eventually, organic impediments to brain function, such as glutamate (Wiehler et al., 2022) or adenosine (Davis et al., 2003) accumulation or interference in cholinergic signaling (Cools and Arnsten, 2022) cannot be compensated by mechanisms that maintain synaptic homeostasis [such as glutamate clearance by astrocytes (Hansson and Rönnbäck, 2004; Rönnbäck and Hansson, 2004)]. These neurometabolic substrates of fatigue guide behavior toward rest; but if a task is highly important, the individual can ignore fatigue feelings and persist in the task despite increased risk of errors.

Described neurobiological mechanisms that contribute to fatigue explain the fact that fatigued individuals display higher reaction times and error rates in a cognitive task. Particularly, the accumulation of adenosine during intense neuronal activity (and given by release of ATP from astrocytes) can negatively affect neuronal conduction velocity (Lezmy et al., 2021); also, adenosine has been implicated in conservation withdrawal behaviors (Plumb et al., 2013). Neurometabolic and conduction velocity alterations in multiple sclerosis, a condition with high prevalence of chronic fatigue, illustrate diverse neurobiological substrates of fatigue (Oliva Ramirez et al., 2021). Regarding acute mental fatigue, accumulation of glutamate could also interfere with neural circuit functions (Wiehler et al., 2022); while there are many mechanisms that contribute to synaptic homeostasis during wakefulness, possibly during prolonged and demanding cognitive tasks, these mechanisms are surpassed. These neurometabolic alterations directly interfere with brain function, sensorimotor processing is slowed, and reaction times are increased (Peters et al., 2022).

Fatigue feelings seem to be informing behavior to preserve organismal homeostasis, guiding the individual to rest, ceasing the task or preserving energy. But given the nature of this feeling, individual perception is subject to distortions (as occurs during disease states like depression). This may be due to distortions in the interoceptive representation of the bodily state, or in agency the system senses over its states. The ability to detect errors and correct them in a changing environment is an essential human ability. From the point of view of the Bayesian theory of the brain, the perception of fatigue in absence of objective fatigue may be given by a discrepancy between the cognitive capacity predicted by the system (according to previous evidence of cognitive performance) and the real cognitive capacity it has. Thus, fatigue feelings may be explained as a discrepancy between previous brain expectations and the information received during the current task. The role of prediction in fatigue perception is reflected in the fact that the knowledge and expectation of task demand affect fatigue sensations (Baden, 2005). The factors that can influence the estimations of this predictive system are diverse; thus, presentation and perception of fatigue are highly variable between individuals. From an evolutionary standpoint, those mechanisms that lead to energy preservation can favor individual biological fitness. Thus, a system that tends to exaggerate physical fatigability may be favorable; but fatigability may have not been subject to the same evolutionary pressures, since sophisticated and prolonged attention tasks such as driving have only recently emerged. The high incidence of fatigue feelings in disease states illustrates a system that seeks to return to homeostasis, to those familiar internal state models it possesses. In contrast to blood osmolarity regulation and the feeling of thirst, currently no sensor of synaptic homeostasis has been detected; feelings of fatigue are not a faithful reflection of real fatigue. It seems that the failsafe in place that allows the return to synaptic homeostasis is sleep par excellence. Future research could help elucidate the relationship between sleepiness and fatigue.

## 5 Limitations

Importantly, subjective fatigue was assessed solely upon the Samn-Perelli scale. Questionnaires that assess different dimensions of perceived fatigue in more depth (and factors that influence this perception, such as personality or social stress) could be useful for future research. No psychophysiological measurements were done, therefore there is no insight on degree of effort deployment, a variable that could highly influence performance (as in compensatory behavior for example). Furthermore, measurements were done in a real-world scenario (before work); variables that may play a role in fatigue development and perception were not controlled for in the study design (such as hours and quality of sleep in the previous night). While this real-world context provides valuable ecological validity, it introduces variability in our results that may obscure interpretation of our findings. Nonetheless, researching fatigue in real-world context provides useful insights for occupational health and contributes to our understanding of fatigue in authentic work environments. Finally, the short duration of the cognitive task in our study could allow for compensatory mechanisms that obscure the assessment of fatigue, but longer durations are not applicable for daily preventive fatigue measurements in the workplace.

## 6 Conclusion

Our findings suggest that SF, measured through the Samn-Perelli scale, is not a good measurement of OF measured through the WAFA Test. This highlights the importance of objective fatigue measurements in the context of fatigue mitigation. Differences in WAFA Test performance between AccessPoint Risk Matrix groups suggest that in severe acute fatigue states, the perception or self-report of fatigue is diminished; and that sometimes SF is present without OF. Together, this evidence speaks about the complexity of the occupational fatigue phenomenon, and the importance of considering that the ability of consciously processing fatigue feelings is highly susceptible to distortion. This is reflected in the Perceptual Blindness to fatigue phenomenon we detected. More research is needed on the factors that influence the conscious experience of fatigue, and for improving fatigue detection systems.

## Data Availability

The raw data supporting the conclusions of this article will be made available by the authors, without undue reservation.
